# Stereological study and analysis of oxidative stress during renal aging in rats^1^

**DOI:** 10.1590/ACTA351106

**Published:** 2020-12-18

**Authors:** Eduardo Felippe Melchioretto, Marcelo Zeni, Djanira Aparecida da Luz Veronez, Francisco Filipak, Ingridy de Souza Digner, Rogerio de Fraga

**Affiliations:** I Fellow PhD degree, Postgraduate Program in Surgical Clinic, Universidade Federal do Paraná, Curitiba - PR, Brazil. Conception and design, manuscript preparation and writing, critical revision.; II Fellow Master degree, Department of Urology, Medical School, Universidade Federal da Fronteira Sul, Chapecó - SC, Brazil. Design.; III PhD, Associate Professor, Department of Anatomy, Medical School, Universidade Federal do Paraná, Curitiba - PR, Brazil. Conception and design.; IV PhD, Associate Professor, Department of Cellular and Molecular Biology, Medical School, Universidade Federal do Paraná, Curitiba - PR, Brazil. Conception and design.; V Graduate student, Little Prince College, Curitiba - PR, Brazil. Design, manuscript preparation and writing, critical revision.; VI PhD, Associate Professor, Department of Urology, Medical School, Universidade Federal do Paraná, Curitiba - PR, Brazil. Conception and design.

**Keywords:** Aging, Kidney, Oxidative Stress, Rats

## Abstract

**Purpose:**

To evaluate renal histological changes by stereology and morphometry and analyze the main markers of oxidative stress in rats undergoing natural aging.

**Methods:**

Seventy two Wistar rats were divided into six groups of 12 rats each, which were euthanized at 3, 6, 9, 12, 18, and 24 months of age. Right kidney was stereologically and morphometrically analyzed to calculate the volumetric density (Vv_[glom]_), numerical density (Nv_[glom]_) and glomerular volume (Vol_[glom]_). Left kidney was used to determine the levels of nonprotein thiols, lipid peroxidation, and protein carbonylation, as well as the activities of superoxide-dismutase and catalase enzymes.

**Results:**

Both Vv_[glom]_ and Nv_[glom]_ values showed gradual decreases between groups. Activity of superoxide-dismutase was elevated at 24 months of age, and the levels of nonprotein thiols were higher in older animals. Greater catalase activity and protein carbonylation were observed in animals between 6 and 12 months of age but lessened in older rats. Lipid peroxidation decreased in the older groups.

**Conclusions:**

Morphometric and stereological analyses revealed a gradual decrease in the volume and density of renal glomeruli during aging, as well as kidney atrophy. These findings related to oxidative stress clarify many changes occurring in kidney tissues during senescence in rats.

## Introduction

Aging is an inevitable process that results in the functional decay of multiple systems, as well as increased morbidity and mortality. Understanding the aging process is currently becoming more important in developing countries due to the accelerated increase in people over 60 years old. In Brazil, which had a population of 210 million in 2018, it was estimated that the number of individuals older than 60 years would reach 32 million in 2020^1^.

In light of this demographic aging and its impact on the human body, it has been shown that the repercussions of kidney senescence are associated with progressive sclerosis, which is linked to a decreased number of functioning nephrons, resulting in decreased renal blood flow and glomerular filtration^2^. This process is accelerated in the presence of comorbidities, resulting in the development of chronic renal failure. The prevalence of chronic renal disease has progressively increased in epidemic proportions in Brazil and throughout the world, creating great concern to governments, due to the high cost of maintaining large patient populations in ongoing renal-replacement therapies^3^.

To appreciate the consequences of this global health issue, it is essential to understand the structural variability that occurs in the kidneys during aging. In this regard, the morphological changes that occur in the kidneys over time are not completely understood, although it is known that they are related to genetic and hemodynamic features. Furthermore, these changes tend to be nonspecific and can be associated with different comorbidities such as diabetes, hypertension, and arteriosclerosis^4^. Stereology can be used to more precisely recognize morphological changes occurring in organs. This method enables the attainment of quantitative data, with minimal bias, through the systematic and uniform analysis of random samples^5^.

Oxidative stress may be related to cellular damage at various levels, such as mutagenesis, carcinogenesis, lipoperoxidation, and protein and carbohydrate oxidation and fragmentation. Therefore, oxidation could also play an important role in the aging process. Reactive oxygen species (ROS) are chemical species with an oxygen-centered free-radical. Reactive oxygen species generation results from univalent reductions in oxygen during aerobic breathing. Thus, all life forms that use this process are subject to the oxidizing effects of reactive oxygen metabolites. Oxidative stress describes a condition with higher levels of ROS, which disrupts the balance between antioxidant cellular defenses and mechanisms that trigger the oxidizing state. Oxidative stress generates compounds that initiate a series of chain reactions involving free radicals, which can oxidize a wide variety of biological molecules^6^.

Fatty acid oxidation in the plasma membrane leads to the destruction of phospholipids and loss of the membrane structure, fluidity, and function. Certain protein side chains contain carbonyl groups, which increase their hydrophilicity and susceptibility to proteolysis. Regarding genetic materials, oxidative reactions may promote the oxidation of macromolecules, such as DNA or the cytoskeletal components, and interfere with the dynamics of the microtubule network of the mitotic spindle, thereby generating genotoxic effects. The presence of ROS can affect the activity of several enzymes, including catalase (CAT), glutathione *S*-transferase, reduced glutathione, glutathione peroxidase, and superoxide dismutase (SOD).

No relevant stereological studies have been performed toevaluate renal structural changes that occur secondarilyto the aging process, with a focus on kidney damage due to concomitant oxidative stress. The method used in this study enabled analysis of these effects during aging, with minimal interference from external conditions, which made it possible to continually monitor the morphological changes that occurred.

The purpose of this study was to examine the renal aging process in rats by evaluating (i) histopathological changes using stereology and (ii) changes resulting from oxidative stress in the renal tissues of animals, with an emphasis on identifying variations in oxidative-damage markers and the main antioxidant defenses in the body.

## Methods

This study adhered to the ethical principles for animal experimentation of the Brazilian College of Animal Experimentation, in addition to the requirements set forth in the *Guide for the care and use of experimental animals*. The experimental protocol was evaluated and approved by the Animal Ethics Committee of the Brazilian College of Animal Experimentation under process number 23075.032620/2010-10.

Seventy-two male Wistar rats (*Rattus norvegicus albinus*) were used in this study. All animals were housed in a controlled environment with a 12-h light/dark cycle and received filtered water and species-specific food *ad libitum* throughout the experiment. They were assigned to six groups, each containing 12 rats, which were euthanized at 3, 6, 9,12, 18, or 24 months of age.

Before the procedure began, the animals were weighed with an ultrasensitive digital electronic scale, Marte brand (model AD1000), maximum capacity 1010 g and minimum 0.01 g. The animals underwent anesthesia via intraperitoneal administration of ketamine hydrochloride solution (57.67 mg/mL)containing 2% xylazine hydrochloride (w/v) at a dose of 1 mL/kg body weight. The surgical procedure consisted of a laparotomy, a median thoracotomy, and a bilateral nephrectomy. To induce cardiorespiratory arrest, an intracardiac puncture was performed, and a sufficient volume of blood was removed to induce fatal hypovolemia. The testicles, bladder, penis, aorta, heart, liver, and brain were also collected for analysis in other studies. For the animal’s height, the nasoanal length was considered, which was measured at the end of the experiment, after the animal’s anesthesia, with the measurements recorded on graph paper.

The left kidney was immediately frozen at –80 °C, and the right kidney was weighed on an analytical balance. The length, width, and thickness of the right kidney were measured using a digital pachymeter (Digimess, manufactured in Chinaand imported by Digimess Ferramentas de Precisão, Ltd.), and,considering the renal ellipsoid pattern, the volume was calculated using the formula recommended by Barr (1990): volume = length × width × thickness × 0.523^7^.

### Histological processing

After taking measurements, each right kidney was fixed for 16 h in ALFAC (80% ethyl alcohol, 40% formaldehyde, and glacial acetic acid; 85:10:5, v/v), which was prepared immediately before use. Next, each kidney was dehydrated in a graded series of xylol and alcohols for subsequent embedding into paraffin blocks. After paraffin embedding, 7-μm thick sections were obtained with a microtome (American Optical, Spencer AO 820, PA, USA). As the cortical portion of the kidney contains the highest concentration of glomeruli, the organ is considered to be anisotropic. Therefore, the orientator method was used to obtain independent uniform random sections^8^.

**Figure 1:**
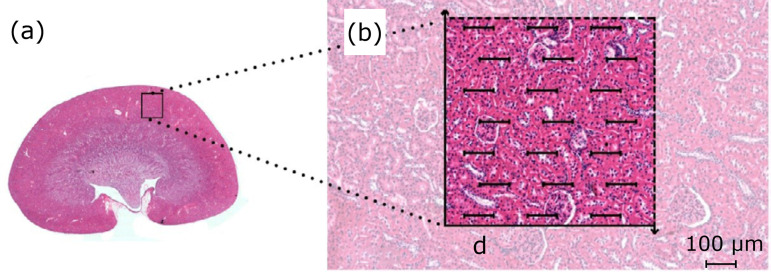
Microscopic image of rat kidney. The renal parenchyma has a single pyramid and consequently a single papilla; **(b)** M42 test system superimposed on the renal cortex. Some glomeruli are touched by the test points and then included in the glomeruli count. *Distance “d” = 100 µm;× 40 magnification.

The physical dissector method was used to calculate the numerical density of the glomeruli (Nv_[glom]_)^9^. Two renal tissues were sampled by selecting one in every five sections (representing a thickness of 35 µm between planes), with the aim of using the two-dimensional sections to quantitatively determine the three-dimensional parameters of the renal glomeruli. Subsequently, five sections from each renal tissue were arranged on the histological slides for hematoxylin and eosin (HE) staining (Fig. 1a). The HE-stained slides were analyzed using a trinocular biological microscope coupled with a BIO2 Polaris microscope (Bel Photonics do Brasil, Ltd., São Paulo, Brazil) equipped with a plain objective, which was connected with a BIOCAM CMOS SERIES 3.0 image-capture system (ZKTeco, China), using TSView 7.1.1 software (Xintu Photonics Co., Ltd., China). From the images obtained, a stereological evaluation of the renal glomeruli was performed to determine the volumetric density (Vv_[glom]_), Nv_[glom]_, and average volume (Vol_[glom]_) of each section of renal glomeruli. The stereological method M_42_ Test System was used to view each three-dimensional kidney image, which was overlaid with the histological images to count the points and test intersections, as described by Mandarim-de-Lacerda^10^ (Fig. 1b).

Equation 1 (in µm^0^) was used to determine the *Vv*_[glom]_ of the renal glomeruli, where *P*_[glom]_ is the number of points crossing the renal glomeruli, and *PT* is the number of test points. The obtained values were expressed as percentages.

$$Vv_{\left[glom\right]}\;=\;P_{\left[glom\right]}/PT$$ (1)

The *Nv*_[glom]_ values of the renal glomeruli were calculated using Eq. 2 (in mm^3^), where *Vol*_[dissector]_ is *e* × *At*, with *e* being the thickness (in µm) and *At* the tested area of the upper plane, and *ΣQ*-_A[glom]_ is the total number of renal glomeruli.

$$Nv_{\left[glom\right]}\;=\;\Sigma QA_{\left[glom\right]}/Vol_{\left[dissector\right]}$$ (2)

The mean volume of renal glomeruli, *Vol*_[glom]_, was calculated using Eq. 3 (in µm^3^), where *Vv*_[glom]_ is the volumetric density and *Nv*_[glom]_ is the numerical density.

$$Vol\left[glom\right]\;=\;Vv\left[glom\right]/Nv\left[glom\right]$$ (3)

### Tissue oxidative stress analysis

The left kidney from each animal was thawed, and the collected tissues were homogenized with frozen phosphate-buffered saline (PBS) and centrifuged at 12,000 × g for 20 min at 4 °C.

Each supernatant was transferred to a separate 2-mL Eppendorf tube for the analyses described below, which were performed through a partnership with the Cell Toxicology Laboratory of the Department of Cell Biology of the Federal University of Paraná.

### Evaluation of lipid peroxidation

The Fox method was used to quantify the concentrations of kidney lipid peroxidase (LPO). The method involves the rapid oxidation of Fe^2+^ (mediated by peroxides under acidic conditions) and subsequent formation of a Fe^3+^–xylenol orange complex in the presence of the stabilizer butylated hydroxytoluene (BHT), which absorbs light at 570 nm. Tubes containing 200 μL of each sample were mixed with 800 μL of reaction medium [100 µm xylenol orange, 25 mM H_2_SO_4_, 4 mM BHT, and250 µm Fe(NH_4_)_2_(SO_4_)_2_·6H_2_O (ammonium ferrous sulfate); added to PA methanol in the described sequence]. After incubation for 20 min at 30 °C, the samples were centrifuged again at 9,000 × g for 10 min. Then, 200 μL of each supernatant was added to a microplate, and the absorbance was measured at 570 nm. The absorbance measurements are expressed as µmols of hydroperoxides/mg of protein^11^,^12^.

### Evaluation of protein carbonylation (PCO)

Protein carbonylation (PCO) was quantified using a model previously described by Levine *et al*.^13^, which is based on the reaction of 2,4-dinitrophenylhydrazine (DNPH) with carbonylated proteins that forms a complex that can be detected by measuring the absorption in the range from 358 to 370 nm. The same quantity of protein in each sample (2 mg) was analyzed. Tubes containing 200 μL of each sample (normalized to the same quantity, i.e., 2 mg) were mixed with 500 μL of DNPH (10 mM of DNPH in 2 M of HCl). To perform the white control (in which no light was absorbed), a separate aliquot of each sample was mixed with an HCl solution (2 M, without DNPH), and the contents were vortexed and stored at 30 °C for 1.5 h. Proteins were precipitated by adding 1 mL of 28% trichloroacetic acid to each sample, followed by centrifugation at 9.000 × g for 10 min. The protein pellets were washed twice by resuspending them in ethanol/ethyl acetate (1:1), vortexing, and centrifugation. Then, with the aim of removing insoluble substances, the proteins were solubilized in 6 M guanidine chloride, and the samples were centrifuged at 9.000 × g for 3 min. Afterward, 200 μL of each sample was transferred to a microplate, and the presence of carbonyls was measured spectrophotometrically at 360 nm. Standard curves for bovine serum albumin (in the presence or absence of DNPH) were constructed and used for PCO determinations, which are expressed here as the number of µmols of carbonyl radicals/mg of protein.

### Dosages of glutathione and other thiols

Fifty microliters of trichloroacetic acid solution (50%) was added to 200 μL of each supernatant or 200 μL of PBS (control). The samples were then centrifuged at 5,000 × g for 10 min at 4 °C. In a microplate, 50 μL of each sample was pipetted into triplicate wells, after which Tris-base buffer (0.4 M, pH 8.9) containing 2.5 mM 5,5-ditiobis-(2-nitrobenzoic acid) was added to each well. The plate was incubated for 5 min at 30 °C, and the absorbance was measured at 415 nm to calculate the glutathione content by comparison with a standard curve. The measurements are expressed as µmols of nonprotein thiols/mg of protein^14^.

### Superoxide dismutase (SOD) activity

The method proposed by Crouch *et al*.^15^ was used to quantify SOD activities. This method depends on the ability of SOD to inhibit the reduction of nitroblue tetrazolium (NBT) to blue formazan by O^2-^, which is generated by hydroxylamine in alkaline solution. The NBT reduction was spectrophotometrically measured at 560 nm. After determining the protein concentrations, each sample was normalized to 2.0 mg/mL. To each tube containing 200 μL of normalized sample, 50 μL of ethanol was added. The contents were immediately centrifuged at 12,000 × g for 20 min at 4 °C. Then, 10 μL of each sample supernatant (analyzed in triplicates) was pipetted into separate wells of a 96-well microplate. As a white control, 10 μL of 25% ethanol were pipetted into PBS in separate wells of the microplate. Next, 20 μL deionized water was added to each well. Then, 70 μL of NBT solution containing 0.05 mM ethylenediaminetetraacetic acid (EDTA) and 100 μL of hydroxylamine solution (36.85 mM) were added to start the reaction. The absorbance at 560 nm was measured at 1-min intervals for 30 min. The measurements are expressed in terms of the activity units/mg of protein. One activity unit is defined as the enzymatic capacity needed to inhibit NBT reduction by 50% (based on a standard curve) and was obtained using the following calculation: (|final absorbance of the standard – initial absorbance of the standard|)/[2 × (|final absorbance of the sample – initial absorbance initial of the sample|)].

### Catalase activity

The method developed by Aebi, was used to quantify CAT activity^16^. This method is based on a decrease in the absorbance at 240 nm due to the degradation of hydrogen peroxide into oxygen and water. After determining each protein concentration, the samples were normalized to 1.0 mg/mL. In a quartz bucket, 990 μL of reaction medium was added, which was prepared by mixing 2.5 mL Tris-HCl buffer (1.0 M) with 5.0 mM EDTA (pH 8.0), 47.32 mL deionized water, and 180 μL H_2_O_2_ (30%, density = 1.1 g/mL, molecular weight: 34 g/mol, final concentration: 30 mM). Then, 10 μL of the sample was added to the cell and mixed by inversion, after which the absorbance was immediately measured spectrophotometrically at 240 nm at 1-s intervals for 60 s. As a white control, only the reaction medium was used. The measurements are expressed as µmols of degraded H_2_O_2_/mg protein.

### Statistical analysis

The data generated during this renal morphometric and stereological study were analyzed by determining the means, medians, minimum values, maximum values, and standard deviations. Analysis of variance was used to test for the effect of life span (3, 6, 9, 12, 18, and 24 months) onthe results related to oxidative stress, and the absolute PCO, LPO, nonprotein thiol, CAT activity, and SOD levels were determined using the Tukey–Kramer test for paired post-hoc comparisons between different groups. Initially, the dependent variables were analyzed in terms of normality, using the Shapiro–Wilks statistical test. The PCO data were normalized by performing a log transformation. However, the mean glomerular volume (Vol_[glom]_) was not normal. Thus, a nonparametric Kruskal-Wallis test was used to test for the effect of the life span on the mean Vol_[glom]_, and a Wilcoxon post-hoc test was performed for pairwise comparisons between the groups. The data were analyzed using SAS JMP Statistical Discover software, version 10.0. The threshold for statistical significance level was set at 95% (p < 0.05).

## Results

**Figure 2:**
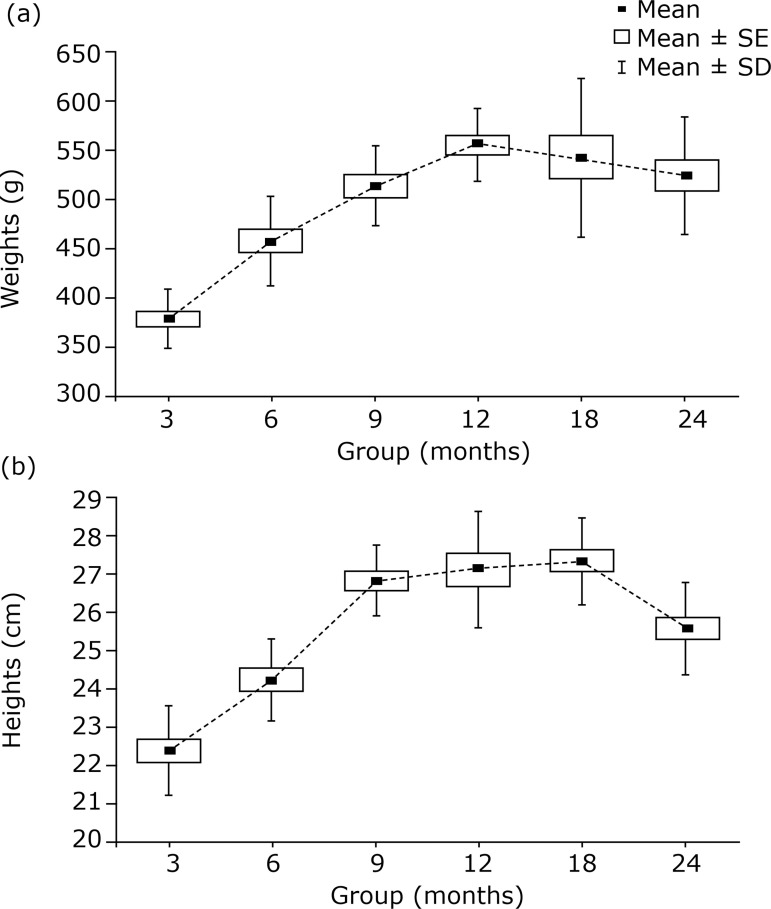
**(a)** Animal weight progress (g) in the different groups; **(b)** Animal height progress (cm) in the different groups (mean, standard error and standard deviation).

As the rats aged during this study, they started to show changes compatible with the aging process, including a reduction in general activity and increased of body hair rarefaction, which was mainly seen in the animals of the 24-month-old group. The weight and length progressively increased between the groups with increasing age, up to 12 months of age, after which a slight decrease in these parameters followed (Fig. 2). No unexpected deaths occurred during the study.

### Renal morphometric analysis

**Figure 3:**
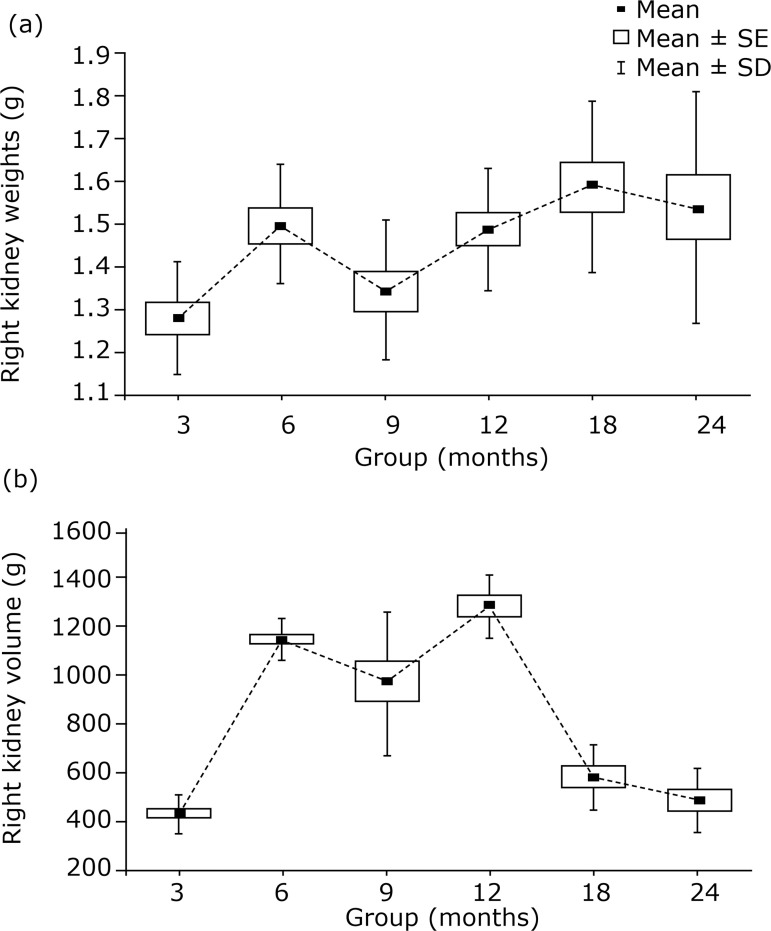
**(a)** Progress of the right kidney weight (g) in the different groups; **(b)** Progress of the right kidney volume (mm^3^) in the different groups (mean, standard error and standard deviation).

Kidney weight analysis revealed statistically lower values in the 3-month-old group (mean 1.28 ± 0.13 g) compared to the other groups (in which the mean ranged from 1.49 to 1.59 g), except for the 9-month-old group, which presented higher values, although they werenot statistically different (mean 1.34 ± 0.16; Fig. 3a). A least-significant-difference (LSD) test revealed a statistically significant increase in the renal volume between the 3-month-old (mean of 433.8 ± 74.58 mm^3^) and 6-month-oldgroups (mean of 1124.6 ± 82.5 mm^3^; p < 0.001). In addition, analysis of the 12-month-old group showed a significant increase in the renal volume compared to the 6-month-old group (mean 1267.2 ± 124.0 mm^3^;p = 0.028). After a peak in the 12-month-old group, the renal volume progressively decreased, and a significant decrease was observed when comparing the 18-month-oldgroup (mean 579.1 ± 137.9 mm^3^; p < 0.001) with the 12-month-old group (Fig. 3b).

### Stereological analysis

**Figure 4:**
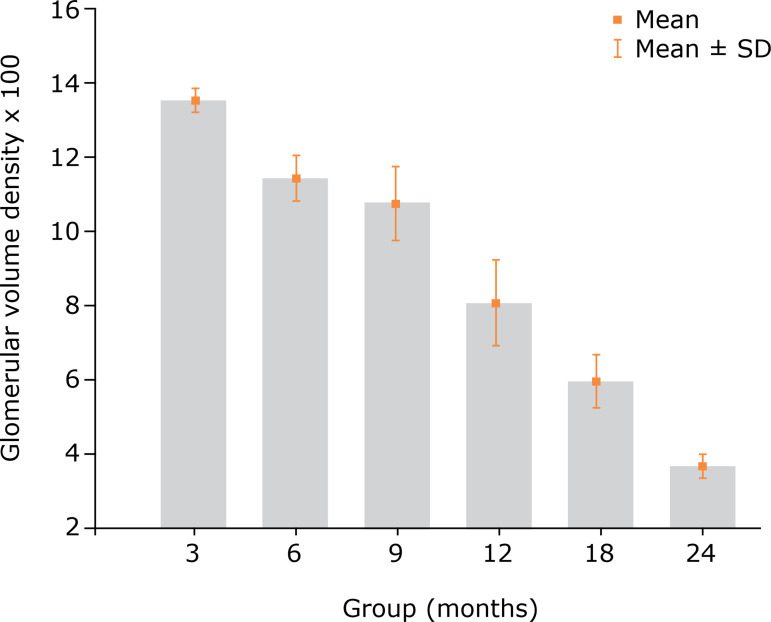
Renal glomeruli volume density (Vv_[glom]_) values in the different groups (mean and standard deviation).

Regarding the Vv_[glom]_ values, a progressive and statistically relevant decrease was found between the groups (Tables 1 and 2, Fig. 4).

**Table 1:** Vv_[glom]_ values in each group (values expressed in µm^0^ × 100; obtained values were expressed as percentages).

Variable	Group	n	Mean	Median	Minimum	Maximum	Standard deviation	p-value*
Vv_[glom]_	3 months	12	13.18	13.26	12.62	13.71	0.37	
	6 months	12	10.72	10.81	9.38	11.81	0.66	
	9 months	12	9.94	10.24	7.81	11.91	1.15	
	12 months	12	6.60	6.41	5.24	9.38	1.32	
	18 months	12	4.50	4.36	3.10	5.62	0.83	
	24 months	12	1.82	1.67	1.43	2.43	0.35	< 0.001

* Single-factor ANOVA, p < 0.05

**Table 2:** Pair-wise values (Vv_[glom]_), (Nv_[glom]_) and (Vol_[glom]_) in the groups.

*Vv*_[glom]_			*Nv*_[glom]_			*Vol*_[glom]_	
Pair-wise group	p-value*		Pair-wise group	p-value*		Pair-wise group	p-value*
3 × 6	< 0.001		3 × 6	< 0.001		3 × 6	0.792
3 × 9	< 0.001		3 × 9	< 0.001		3 × 9	< 0.001
3 × 12	< 0.001		3 × 12	< 0.001		3 × 12	< 0.001
3 × 18	< 0.001		3 × 18	< 0.001		3 × 18	< 0.001
3 × 24	< 0.001		3 × 24	< 0.001		3 × 24	< 0.001
6 × 9	0.029		6 × 9	0.007		6 × 9	0.007
6 × 12	< 0.001		6 × 12	< 0.001		6 × 12	< 0.001
6 × 18	< 0.001		6 × 18	< 0.001		6 × 18	< 0.001
6 × 24	< 0.001		6 × 24	< 0.001		6 × 24	< 0.001
9 × 12	< 0.001		9 × 12	< 0.001		9 × 12	0.144
9 × 18	< 0.001		9 × 18	< 0.001		9 × 18	0.457
9 × 24	< 0.001		9 × 24	< 0.001		9 × 24	0.178
12 × 18	< 0.001		12 × 18	< 0.001		12 × 18	0.467
12 × 24	< 0.001		12 × 24	< 0.001		12 × 24	0.006
18 × 24	< 0.001		18 × 24	< 0.001		18 × 24	0.039

* LSD test

**Figure 5:**
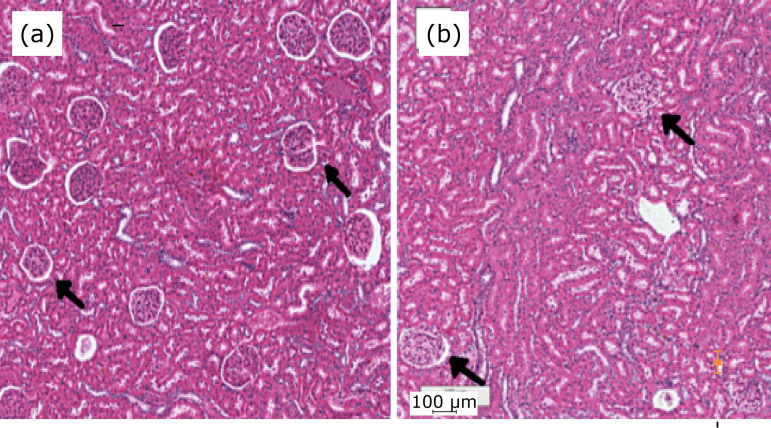
Photomicrograph of the kidney cortex using hematoxylin-eosin. Magnification: × 40. Marked difference between the number of identified glomeruli (*arrows*) in the **(a)** 3-month group *vs*. **(b)** 24-months group.

**Figure 6:**
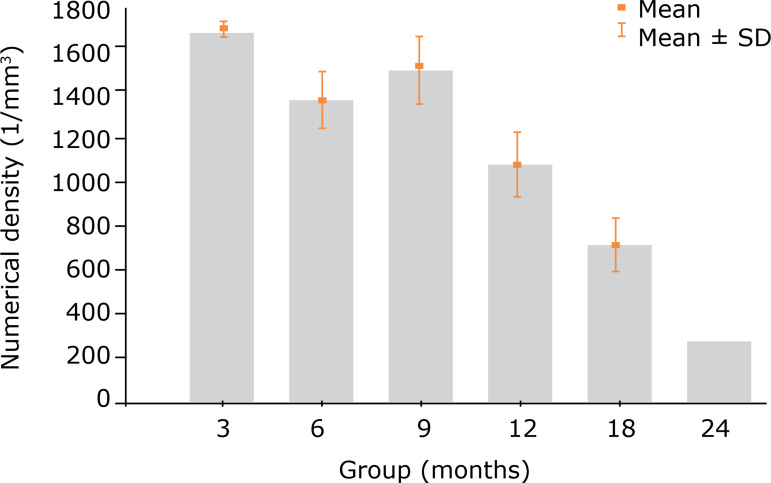
Renal glomeruli numerical density (Nv_[glom]_) values in the different groups (mean and standard deviation) (values in 1/mm^3^).

Quantitative density measurements, in terms of the Nv_[glom]_ values, showed a statistically significant decrease between rats at 3 and 6 months of age (p < 0.001). In addition, compared with the 6-month-old rats, the animals at 9 months showed an increase in this variable (p = 0.007). The Nv_[glom]_ of the renal glomeruli in the groups after 9 months of age showed, then, a progressive decrease until reaching the group of rats at 24 months of age. This progressive decrease showed a strong statistical signal (p < 0.001) (Table 3, Figs. 5 and 6).

**Table 3:** Renal glomeruli numerical density (Nv_[glom]_) values in the groups (values expressed as the number of renal glomeruli/mm^3^).

Variable	Group	n	Mean	Median	Minimum	Maximum	Standard deviation	p-value *
Nv_[glom]_	3 months	12	1657.7	1662.1	1604.4	1717.9	31.3	
	6 months	12	1353.2	1351.0	1138.5	1608.9	128.9	
	9 months	12	1487.1	1508.2	1250.0	1771.9	157.8	
	12 months	12	1059.5	1063.1	799.1	1283.2	147.5	
	18 months	12	694.9	655.5	461.0	927.5	124.4	
	24 months	12	264.2	242.3	207.6	353.0	50.7	< 0.001

* Single-factor ANOVA, p < 0.05

**Figure 7:**
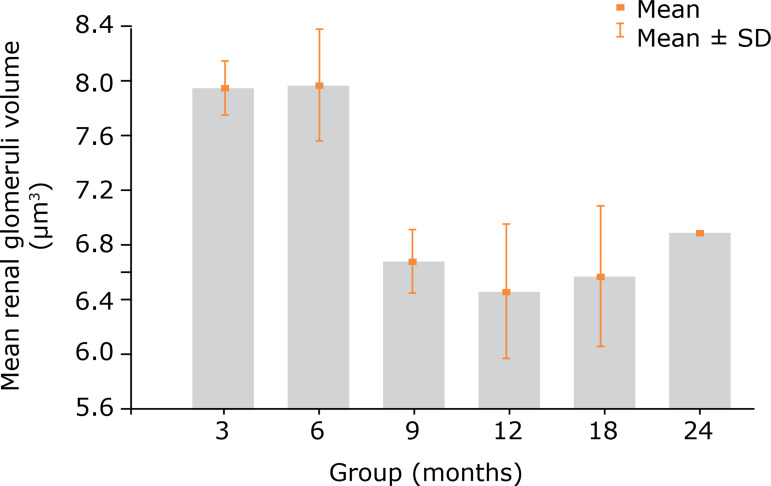
Mean renal glomeruli volume (Vol_[glom]_) in the different groups (mean and standard deviation). (values µm^3^).

The mean Vol_[glom]_ values showed no statistical difference between rats aged 3 or 6 months (p = 0.792). Regarding the 6-month-old group, a statistically significant decrease (p < 0.001) was observed in the mean Vol_[glom]_, compared with that in the other older groups. Evaluating this variable in rats older than 9 months revealed no significant variations (Table 4, Fig. 7).

**Table 4:** Vol_[glom]_ values in each group (values expressed in µm^3^).

Variable	Group	n	Mean	Median	Minimum	Maximum	Standard deviation	p-value*
Vol_[glom]_	3 months	12	7.96	7.99	7.67	8.26	0.20	
	6 months	12	8.00	8.03	7.19	8.58	0.40	
	9 months	12	6.68	6.73	6.22	7.08	0.24	
	12 months	12	6.45	6.48	5.83	7.37	0.51	
	18 months	12	6.56	6.66	5.37	7.26	0.54	
	24 months	12	6.88	6.88	6.88	6.88	0.001	< 0.001

* Single-factor ANOVA, p < 0.05

### Tissue oxidative stress analysis

#### Oxidative markers

**Figure 8:**
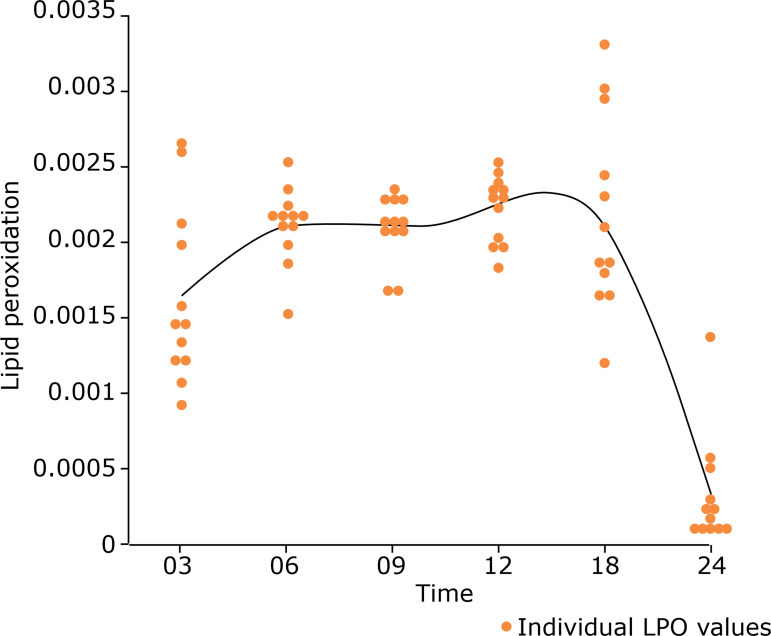
Lipid peroxidation values in the different groups. Measurement: µmols of hydroperoxides·mg·prot^-1^.

Analysis of the LPO results using a paired-Wilcoxon test showed a significant reduction in lipid peroxidation (p < 0.0001) at 24 months compared with that in the other groups in which the analysis of renal tissues presented similar values for this variable in the younger groups (p-values ranging between 0.0193 and 0.8852; Fig. 8).

**Figure 9:**
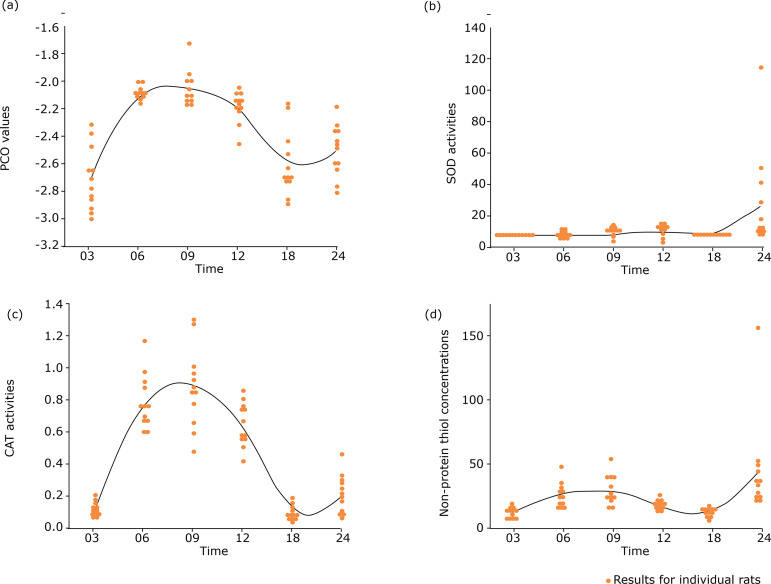
**(a)** Protein carbonylation values in the groups (measurement: µmols of carbonyl radicals·mg·prot^-1^). **(b)** Superoxide dismutase (SOD) activity values in the groups (measurement: activity units·mg·prot^-1^). **(c)** Catalase activity values in the groups (measurement: µmols of degraded H_2_O_2_·mg·prot^-1^). **(d)** Nonprotein thiol concentration in the groups (measurement: µmol nonprotein thiols·mg·prot^-1^).

Protein carbonyl group levels were analyzed using a Tukey-Kramer test, which did not show uniform or significant increases among all groups. However, the number of carbonyl groups in proteins was statistically higher (p < 0.0001) in the renal tissues of animals at 6, 9, and 12 months of age than in rats from the other groups (where the p-values ranged from 0.0134 to 0.9998; Fig. 9a).

##### Antioxidant factors

Analysis of the SOD enzyme activity using a paired-Wilcoxon test showed statistically higher values (p < 0.05) in the renal tissues of older animals (24 months of age), compared to those in the younger groups (Fig. 9b).

Catalase enzyme activity, when analyzed by the Tukey–Kramer test, did not show uniform or significant increases among all groups (considering that the p-value ranged from 0.176 to 0.9997). Similar CAT activities were observed in the groups at 3, 18, and 24 months of age (considering that the p-value ranged from 0.3883 to 0.9997) and similar CAT activities were also observed in the groups at 6, 9, and 12 months of age (considering that the p-value from 0.0026 to 0.1768). However, a comparison of rats of intermediate age (6 to 12 months) with older animals (18 and 24 months of age) showed a significant reduction in CAT activity (p < 0.0001; Fig. 9c).

The biochemical dosages of nonprotein thiols analyzed by the Tukey–Kramer test did not show uniform or significant increases among all groups (considering that the p-value ranged from 0.0835 to 1.0000), although a significantly higher concentration (p = 0.0002) was found when comparing the dosages between the 3- and 24-month-old groups (Fig. 9d).

## Discussion

The main objective of this study was to analyze the impact of aging on the kidneys of rats living in a controlled environment. Morphometric and stereological analyses revealed kidney atrophy throughout senescence, as well as progressively decreasing Vol_[glom]_ and Nv_[glom]_ values during aging. In addition, the SOD activity increased in senile rats, which was associated with higher levels of non-protein thiols in older animals.

In this study, the rats in the 24-month-old group were considered elderly, based on their known life expectancy^17^. According to previous results, the weight changes observed in this study were considered appropriate^18^.

Several histopathological changes are associated with the renal-aging process, such as organ atrophy, a decreased number of epithelial cells on the contorted tubules, a decreased number and volume of nephrons, arterial changes, and increased numbers of renal interstitial cells and interstitial fibrosis. In this study, renal repercussions associated with aging were evaluated based on morphometry and stereology^5^.

Regarding the renal weight changes observed in rats during aging, Takeda and Alabarse *et al*.^20^ reported data similar to those of the present study, reinforcing the possibility that the renal weights do not significantly vary during the aging process^20^. However, they reported a mean kidney weight slightly higher than that found in this study.

Analysis of the renal volumes showed a gradual increase, peaking at 12 months, followed by a progressive decrease, which demonstrated that the organ underwent atrophy in older animals^21^.

Stereological analysis demonstrated, with strong statistical significance, progressive decreases in the Vv_[glom]_ and Nv_[glom]_ values in the renal tissue of older animals. These findings demonstrate changes that resulted from the renal-aging process. In 1926, Arataki published a study with similar findings, which was based on a simpler method and did not involve the use of stereology^22^.

The data from this study provided evidence that the mean Vol_[glom]_ varied as the rats aged, in a manner that agreed with previous findings in humans^23^,^24^. In the present study, the mean Vol_[glom]_ decreased in animals in the 9-month-old group, which was not reported by Cortes *et al*.^25^. In contrast, Cortes *et al*.^25^ found that the mean Vol_[glom]_ progressively increased in Fischer rats, according to their development. In that scenario, the decline in the number of glomeruli with preserved function possibly resulted in a compensatory hypertrophic response by the remaining glomeruli in the senile rats.

In this study, oxidative damage was identified by the presence of LPO and PCO in the renal tissue of animals of different ages.

Lipid peroxidase analysis showed no clear progressive pattern. A simpler analysis and comparison between the youngest and oldest groups showed a significant reduction of this marker in the oldest group. In contrast to the findings of this study, Arivazhagan *et al*.^26^ showed a significant increase in LPO in old rats that was more consistent with what was expected during aging and was similar to findings reported by Uzun *et al*.^27^.

Protein carbonylation was significantly reduced in the older groups when compared to the 6- and 12-month-old groups, similar to data presented by Alabarse *et al*.^20^. This phenomenon may be related to lower oxygen consumption at an older age, due to a decreased total amount of proteins in the tissues, with glomeruli progressively being replaced by fibrous tissue^11^. Similar results to those reported in this study for renal tissues were previously seen in other organs in male rats during the monitored stages. For example, pulmonary carbonyl levels were higher at 6 months than at 3 and 12 months of age; carbonyl levels in the brain were higher at 6 months than at 3 and 12 months of age, and SOD activity in the brain decreased from 3 to 6 months of age^28^,^29^. As previously mentioned, differences found between organs are related to a multifactorial setting that also involves specificities inherent to the physiology of the organ of interest^29^. However, even though different oxidation patterns occur, resulting in different functional activities, aging imposes a global influence on the body. Over time, similar oxidative processes occur between the different organs.

Analysis of the SOD and CAT activities, as well as the nonprotein thiol levels, showed that the three main antioxidant factors did not display an increasing linear pattern with increasing age.

Thiol groups are reducing agents that can be used as an alternative approach to assess oxidative repercussions. Lower thiol levels are indicative of increased oxidative stress^30^.

The present findings indicated that the 24-month-old rats (when compared with the 3-month-old group) showed increased SOD activity and higher nonprotein thiol levels. These data corroborate the observation that higher antioxidant activities were found in older animals and could explain why the impact of oxidative damage was not as high as expected. In addition, the animals in the present study remained for a lifetime in a controlled environment and without any added stressful stimulus. This may have determined livelihood conditions more conducive to maintaining adequate antioxidant protective activities. This would be consistent with what Marchon *et al*.^31^ identified in their study, which found suspension of kidney damage in animals that stopped suffering stress stimuli.

Another possible explanation for the findings of increased nonprotein thiol levels and the increased SOD activity in older animals would be the potential for less cellular damage by oxidative stress in already senile kidneys and with lower volumetric density (Vv_[glom]_) and numerical density of the glomeruli (Nv_[glom]_).

In contrast to the animals analyzed in this study, Uzun *et al*.^27^ found that SOD activities were significantly lower in renal tissues from 24-month-old rats than those from 6-month-old rats. Other previous studies revealed no significant difference in SOD activities in renal tissues during the aging process^20^,^32^.

Additionally, in contrast to the current findings, Aydın *et al*.^33^ showed a significantly decreased concentration of non-protein thiols in older animals. The results of other studies did not reveal relevant concentration differences of nonprotein thiols in animals at different ages^27^,^34^.

Regarding the CAT enzyme activity in the rat renal tissues, the analysis of this study showed similar enzyme activities among animals of intermediate age (6-12 months) and decreasing CAT activities in older animals (18-24 months of age). The decreased CAT activity found during the aging process is consistent with data from other studies, such as that conducted by Gündüz *et al*.^32^ In that study, they investigated the role of physical exercise in Wistar rats by reducing the repercussions caused by oxidative stress and identified (in the group without intervention) a 45%-decrease in CAT activities in the renal tissue of 24-month-old rats when compared to 9-month-old rats.

Although no linear relationship was seen between the antioxidant factors, it can be inferred that there was an increase in nonprotein thiols and SOD activities, which compensated for the decreased CAT activities. This possibility is corroborated by the reduction of the main antioxidant pathway (CAT), with similar or decreasing increasing levels of oxidative-damage markers (LPO and PCO) in the older group.

Previous groups have reported inconsistent findings in terms of the impact of aging on antioxidant enzymes^35^–^37^. In addition, with aging, the performance of these enzymes generally undergoes modifications with varied forms of evolution according to different tissues^38^.

The data found in the present study showing renal atrophy, glomerular loss and some changes in antioxidant enzymes in older rats. This could infer that aging predisposes to kidney injury, but laboratory tests of kidney function would be necessary to be able to determine that these findings would determine some degree of renal dysfunction.

## Conclusions

Morphometric and stereological analyses showed that significant renal atrophy occurred during aging, which was associated with progressive reductions in the Vol_[glom]_ and Nv_[glom]_. However, no compensatory glomerular hypertrophy was found in senile rats.

Protein carbonylation levels and LPO activities decreased in the renal tissues of older animals. In addition, CAT enzyme activities were reduced, and nonprotein thiol levels and SOD activities increased in the older animals compared with the younger animals.

Although it cannot be concluded that these changes represent the cause of cellular aging, the study’s findings related to oxidative stress reveal many changes in kidney tissues during the senescence process in rats.
